# HIV Viral Re-Suppression on Second-Line ART in Southern Zimbabwe

**DOI:** 10.3390/ijerph22050730

**Published:** 2025-05-02

**Authors:** Kudakwashe Musomekwa, Brian van Wyk

**Affiliations:** 1 AIDS Healthcare Foundation, Mpilo Centre of Excellence, 2096 Vera Road, Mzilikazi, Bulawayo VHF9+2XR, Zimbabwe; 4104720@myuwc.ac.za; 2 School of Public Health, University of the Western Cape, Cape Town 7535, South Africa

**Keywords:** HIV, viral suppression, viral load, retention in care, antiretroviral therapy

## Abstract

The national prevalence of HIV among adults in Zimbabwe was 10.4% in 2023, while the HIV prevalence in Bulawayo Metropolitan Province was 11.7%. The country achieved the UNAIDS Fast Track goals of 95-95-95 ahead of the 2030 target, by reporting an ART coverage of 97.0% and a viral suppression rate of 95.0%. As the number of people on ART continues to grow, it is expected that the number of patients failing current first-line non-nucleoside treatment will increase. However, access to second-line treatment regimens remains very limited in resource-poor settings such as Zimbabwe. It is therefore imperative to review treatment success for persons on second-line treatment. A retrospective cohort analysis was conducted using routinely collected clinical and demographic data from 315 participants who had been on a second-line ART regimen in Bulawayo for at least six months between 2015 and 2020. Statistical analyses were conducted to identify risk factors for viral suppression using SPSS version 28. Viral suppression (68.6%) for adults was far below the target of 95%. After accounting for all other variables, baseline CD4 count (>200 c/µL) [AOR = 1.94 (1.05–3.61)], having no history of non-adherence on first-line ART [AOR = 3.88 (1.91–7.85)], drug switch within 12 months of failure [AOR = 4.13 (1.98–8.60)] and retention in care at 5 years [AOR = 6.35 (2.56–15.76)] predicted viral re-suppression. The second-line (rescue) regimen has not achieved the expected success in reversing initial viral non-suppression (due to late presentation and poor adherence), despite enhanced adherence counselling. Timely switching is effective when done within 12 months and coupled with persistent engagement in care. We recommend improved methods, such as enhanced adherence counselling for behaviour change to improve viral re-suppression rates, especially for those on with a history of poor adherence and virologic non-suppression.

## 1. Introduction

The national prevalence of HIV among adults in Zimbabwe was 10.4% in 2023, with an HIV prevalence of 11.7% in Bulawayo Metropolitan Province [1]. Approximately 1,300,000 adults were living with HIV in Zimbabwe in 2023 and the country has achieved some of the UNAIDS Fast Track goals of 95-95-95 by 2030, with reports of ART coverage of 97.0% and a viral suppression rate of 95.0% [2,3].

As the number of people on ART continues to grow, it is assumed that the number of patients failing current first-line non-nucleoside reverse transcriptase inhibitor (NNRTI)-based antiretroviral regimens (ARV) and needing second-line protease-inhibitor (PI)-based ARVs will increase [4,5]. However, access to second-line treatment regimens remains very limited in resource-poor settings such as Zimbabwe [6]. Furthermore, there is a paucity of evidence on the efficacy of second-line ART in many countries in Sub-Saharan Africa [7,8]. The effectiveness of ART programmes is generally measured in terms of rates of viral suppression and retention in care, as recommended by the World Health Organisation (WHO) [9]. However, there is limited evidence on the effectiveness of second-line ART in Sub-Saharan Africa in terms of viral re-suppression [8]. It has been demonstrated that continuous engagement on a PI-based second-line ARV regimen is more difficult due to the higher pill burden and increased toxicity associated with the drugs [5]. Since it is well-established that retention in ART is associated with viral suppression, these primary outcomes are critical in monitoring the successes of HIV programmes [10,11]. It is imperative that determinants of viral re-suppression be documented to enable evidence-based clinical management of patients on second-line ART.

**Figure 1:**
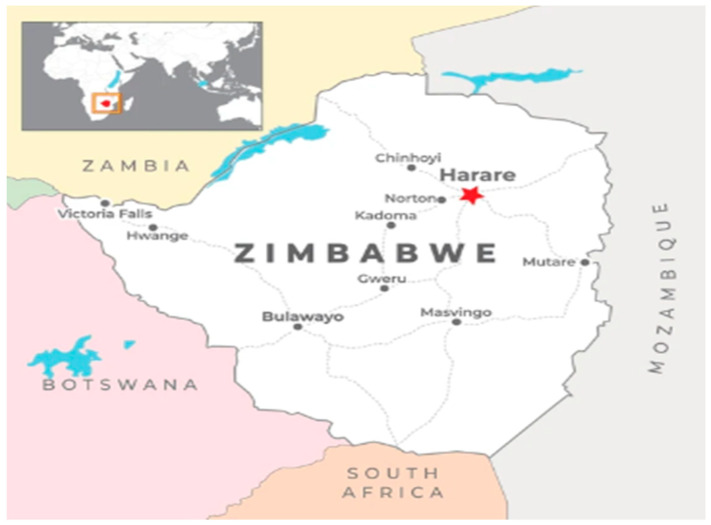
Study site location of Mpilo Centre of Excellence, Bulawayo.

Zimbabwe has four specialised clinics dedicated to HIV treatment, with three located in the capital city of Harare and one in Bulawayo, which is the country’s second-largest metropolis. These facilities serve as referral centres and are staffed by specialists and equipped with comprehensive laboratory and radiology support [5]. This is commendable given that laboratory and clinical capacity within Sub-Saharan Africa remains limited [12]. Mpilo Centre of Excellence (COE) is the largest ART clinic in Zimbabwe (Figure 1); serving an estimated 11,500 patients on ART in 2018 [13]. The clinic was a pioneering ART clinic in 2004 when the Ministry of Health (MOH) began ART rollout in the public sector [13]. Ever since that time, both coverage and long-term ART management have grown with the years, and the maturation of the ART cohort has led to an increase in the number of patients in need of second- and third-line treatment. The clinic, as of June 2021, had 1768 clients on second-line ART—translating to 15% of the total number of patients.

Drug-resistance testing for the selection of antiretroviral drugs for second-line ART is not routinely available in the public sector in Zimbabwe. Therefore, patients at the clinic are only switched from first-line therapy following the MOH National ART guidelines. The last-mentioned guidelines indicate a comprehensive package of care that includes laboratory and clinical parameters where patients with elevated viral load (VL) levels of >1000 copies/mL are referred to counsellors for enhanced adherence counselling (EAC) as per the standard of care [14]. A patient receives three EAC sessions in three months followed by a repeat VL test. If the VL result remains 1000 copies/mL or more, the patient is considered to have virological failure and is switched to a second-line ART regimen [15]. The switch to second-line regimens involves the replacement of the NNRTI component with a PI, and with the switch of one or both nucleos(t)ide reverse transcriptase inhibitors (NRTI) from the first-line regimen to NRTIs that the individual has not been exposed to in treatment thus far.

## 2. Materials and Methods

A retrospective, cohort study was conducted to determine the factors associated with VLS among individuals receiving second-line ART. Mpilo Centre of Excellence (COE) was a pioneering referral centre for providing ART in the public hospitals in Zimbabwe in 2004. Mpilo COE is considered a high-volume ART facility with 11,500 registered patients in December 2018 [13]. The clinic is located in the city of Bulawayo in the South-Western part of Zimbabwe, approximately 440 km from the capital city, Harare.

The study population comprised all individuals who were receiving second-line ART in the five-year period between 1 January 2015 and 31 December 2020 (N = 1745). The study sample consisted of individuals who received second-line ART for at least six months from Mpilo COE during the abovementioned period (N = 315).

The data were extracted from an electronic patient management software named Electronic Point of Care (EPOC). The EPOC software collates routine clinical data collected from all the departments (pharmacy, laboratory, clinical and counselling) and it stores an electronic record of all the clients receiving care at the clinic. The EPOC software, the national ART registration book and the patients’ hospital booklets were utilised to collect information on patients who had received second-line ART between January 2015 and December 2020. Data quality checks were achieved through triangulation of the above data sources to minimise data incompleteness and inconsistency.

The primary outcome variable was viral load suppression: a VL < 1000 copies/mL was considered ‘suppressed’ and a VL > 1000 copies/mL was considered ‘unsuppressed’. These VL cut-off points were in accordance with the national HIV treatment guidelines in Zimbabwe at the time of the study [14,15].

Demographic variables included gender, age, age at ART initiation, marital status, employment status and educational status. Clinical characteristics included the baseline CD4 count and viral load, the World Health Organisation stage, the first- and second-line ART drug regimens, history of drug switch and poor adherence on first-line ART, duration on first-line ART, time between failure and switch to second-line ART, number of enhanced adherence counselling sessions and baseline body mass index.

Firstly, a descriptive analysis of the data was conducted whereby the completed structured data extraction checklists were transferred to a Microsoft Excel 2020 spreadsheet for cleaning and coding and afterwards exported to SPSS version 28 for analysis. Bivariate analysis was conducted on clinical and demographic factors by viral suppression and tested using chi-square tests with significance set at *p* < 0.05. The significant factors were then uploaded into a multi-variable model leading to the computation and identification of factors independently associated with viral re-suppression. These were presented as adjusted odds ratios (AOR) with 95% confidence intervals (95% CI).

Ethical clearance was obtained from the Mpilo COE Ethical Review Board, The University of Western Cape Biomedical Research Ethics Committee (ref. no. BM22/9/10) and The Medical Research Council of Zimbabwe (ref. no. MRCZ/B/2541). Protection of personal information was attained by ensuring that no names of participants or any identifying data were used, and no personal identifying information such as patient name/surname or identity number was extracted from the database or during the study. This study was conducted in compliance with the 1964 Declaration of Helsinki guidelines and its subsequent amendments.

## 3. Results

### 3.1. Realisation of the Sample

**Figure 2:**
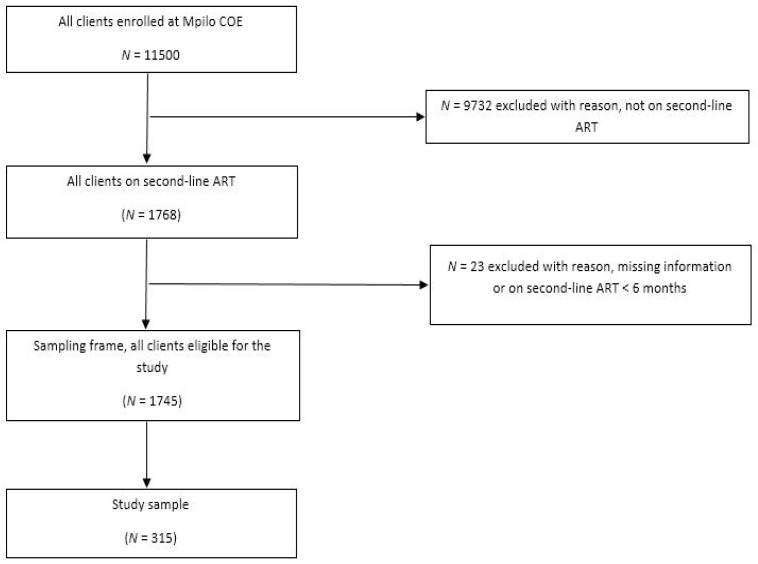
Flow chart giving an outline of the study’s sample realisation.

Mpilo COE had an enrolment of 11,500 registered patients as of December 2018 [13]. Of these enrolled clients, 1768 clients were on second-line ART, which translates into 15% of the total number of patients (Figure 2). A total of 9732 clients were excluded from this study because they were not on second-line ART and an additional 23 clients were excluded because they had missing baseline information or they had been on second-line ART for fewer than six months on 1 January 2015. Hence, a total of 1745 clients were eligible to be included in the study sample.

### 3.2. Characteristics of Study Participants

There were almost an equal number of females (50.2%; n = 158) and males (49.8%; n = 157) as participants in the study (Table 1). The majority of the study participants older than 18 years (n = 272) were single (63.6%; n = 173) and unemployed (82.7%; n = 225). The age of the participants ranged from seven to 84 years old with a median of 30 years and a mean of 34 years. Most of the study participants were classified as adults aged over 24 years (62%; n = 196) and had received at least a primary education (69.5%; n = 219), with fewer (30.5%; n = 96) reporting no formal education.

Slightly more than half of the study participants (54.6%; n = 172) had advanced HIV disease, classified as World Health Organisation (WHO) clinical stages III and IV, whereas most participants had viral loads (VL) greater than 5000 copies/mL (72.4%; n = 228). Furthermore, out of the 315 study participants, a total of 233 (74.0%) and 213 (67.6%) participants had a BMI ≥ 18.5 kg/m^2^ and CD4 count greater than 200 cells/µL at second-line ART initiation. D4T-3TC-NVP (43.3%; n = 152) and ABC-3TC-ATZ/rtv (25.7%; n = 81) were the most prescribed first and second-line antiretroviral regimens.

Concerning medication adherence and drug substitution history on first-line ART, 135 (42.9%) study participants had no history of poor adherence while on first-line ART and 175 (55.6%) participants had no history of drug substitution while on first-line therapy. Fewer than two-thirds of the study participants, 190 (60.3%), experienced delayed switching to a second-line ARV regimen (more than 12 months after confirmed virological failure). Moreover, most study participants (54%; n = 170) were established on ART, having been on treatment for more than five years. At the end of the study period, the majority (83.5%; n = 263) of the study participants were alive and receiving care.

### 3.3. Viral Suppression

Viral suppression (<1000 copies/mL) on second-line ART in this study was 68.6% (n = 216) and retention in care was 83.5% (n = 263). In bivariate analysis, viral re-suppression was significantly associated with having more than six EAC sessions (*p* = 0.010) and a higher baseline CD4 count (>200 c/µL) (*p* = 0.039) (Table 1). Significant associations were found between viral re-suppression and viral load at the point of drug switch (*p* = 0.046) as well as the duration on first-line ART (*p* = 0.005). Viral suppression was higher in those with no documented history of poor adherence (85.9%, n = 116) and were switched to second-line ART in less than 12 months after confirmed virological failure (87.2%, n = 109). Study participants who were retained in care recorded higher viral re-suppression rates compared to those who were not retained (74.1% vs. 40.4%; *p* ≤ 0.001).

In multivariate analysis, viral re-suppression was significantly associated with baseline CD4 count, having no history of non-adherence on first-line ART, time taken between failure and drug switch and retention in care (Table 2). There were no significant associations between viral re-suppression and all the studied demographic factors.

## 4. Discussion

Our study reports viral suppression for participants on the second-line ART regimen at 68.6% and retention in care at 83.5%, which is below the target of 95% viral suppression for patients on ART set by the Joint United Nations Programme on HIV/AIDS (UNAIDS) [16]. According to the 2020 Zimbabwe Population Health Impact Assessment (ZIMPHIA) [4] survey, 97.0% of those diagnosed with HIV reported being on treatment, and the national viral suppression rate for adults who were on ART was 90.3%. Hence, the findings of this study are significantly lower than the Zimbabwe national HIV viral suppression rates. Though VL suppression on first-line ART at the clinic stood at 87%, there was no statistically significant association between viral suppression and either first- or second-line regimens.

A considerable number of similar studies conducted in Sub-Saharan Africa have reported viral suppression on second-line ART above 70% [17,18,19]. Though slightly lower, our findings are similar to a study conducted in Northern Tanzania [20]. This difference might be because the other studies were multicentre studies with bigger sample sizes as compared to the present study. This low suppression rate on second-line ART partly influenced the decision by the MOH to partner a private voluntary organisation to enable access to drug resistance testing targeting individuals failing treatment on second-line ART to enhance clinical decisions in managing these particular clients.

The CD4 count is the most significant laboratory indicator of immune function in individuals living with HIV [21]. Hence, it was once used to determine when to commence treatment in individuals living with HIV before the advent of the World Health Organisation (WHO) treat-all strategy [22]. According to findings from clinical trials and cohort studies, it was also identified as the strongest predictor of disease progression and survival in people living with HIV (PLHIV) [20]. In this study, a baseline CD4 count ≤ 200 cells/µL was presumed to mean late presentation to care. The finding from the current study is similar to what Edessa, Sisay and Asefa [23] reported in their systematic review on second-line HIV treatment failure in Sub-Saharan Africa. Their report found (moderate to high quality of evidence) that low baseline CD4 counts were associated with increased rates of failing to suppress on second-line ART. On the contrary, a cross-sectional analysis conducted in the Ehlanzeni district in South Africa revealed a lower likelihood of viral suppression among adolescents with a higher CD4 count at baseline [24]. Although Okonji et al. [24] investigated viral suppression, this information is useful for also assessing the relationship between CD4 count and viral re-suppression, as was done in the current study.

The benefits of good medication adherence have been widely documented, with studies conducted in both developed and developing countries confirming the strong correlation between good medication adherence and viral suppression [25,26,27]. These findings were confirmed in the present study, where participants who had no history of non-adherence had a significantly higher probability of experiencing viral re-suppression on second-line ART. Furthermore, according to Ramadhani et al. [28], adherence to first-line ART is an important predictor of adherence to second-line ART.

A delayed regimen switch after the first virological failure is associated with failure to re-suppress on second-line ART. This has been confirmed by several studies that have concluded that a delayed regimen switch after the first virological failure is associated with poor treatment outcomes [28,29,30]. In Uganda, delaying a regimen switch to second-line ART for more than 12 months, compared to switching within the first six months following virologic failure, was associated with more than a five-fold increased risk of developing either VL increase, immunologic decline or death [19]. Similarly, staying on a failing regimen was also associated with an increased risk of mortality in Northeast Ethiopia [30]. The Zimbabwe MOH guidelines state that, in the absence of major adherence barriers, switching to a new regimen should be carried out within two weeks of receipt of the second high viral load laboratory results [14]. These guidelines have been made widely available through online platforms and the MOH arranges for periodic continuous medical education sessions for clinicians [14,15]. Though adequate training and ongoing mentorship are mentioned as key facets in the guidelines, ineffective dissemination of the guidelines and training of clinicians in the management of treatment failure was cited as one of the contributing factors to delayed switching to second-line therapy in Zimbabwe [14,31].

In the current study, the patient activity status (retention) was significantly associated with viral re-suppression on second-line ART. This finding has been confirmed by a study conducted in Ethiopia [29]. Retention in care is essential to ensure patients achieve viral re-suppression. Maskew et al. [32] identified retention on treatment and being virally suppressed as the most effective preventative tools for reducing new HIV infections.

This study was a retrospective cohort study that relied on routinely collected patient data during clinical interactions at clinic visits. Hence, the possibility of having incomplete data records, poor data quality or misclassification of participants could potentially have affected the results. The study utilised a hospital-based sample; therefore, the study findings may not be generalisable beyond the study setting. Availability of drug resistance testing would have added valuable insight to the findings of this study. Unfortunately, during the period under review, no participant had an opportunity to undergo drug resistance testing.

However, the study is the first of its kind to be conducted in Bulawayo, Zimbabwe. The analysis of routinely collected clinical data provides a realistic perspective into the HIV service delivery’s strengths and gaps for the HIV population under study, including the information gaps (as reflected by the incomplete data). Our study sample was also all-inclusive of all individuals on second-line ART, which reduced biases due to sampling or selection. However, the study could be enhanced through the use of a mixed-methods approach, whereby qualitative methods are used to investigate in-depth the non-biomedical barriers to adherence among unsuppressed individuals on second-line ART.

## 5. Conclusions

Viral suppression for participants on second-line ART regimens at Mpilo COE stood at 68.6%, which is below the UNAIDS target of 95%. Clinical profiles emerged as the most significant factors leading to low levels of viral re-suppression on second-line ART. We recommend that clinical laboratory diagnostic tests such as CD4 cell count and the HIV drug resistance test be consistently available to enable better management of clients failing treatment. Refresher courses for clinicians on the management of virological failure and timely switching of drugs, coupled with training of counsellors on enhanced adherence counselling and retention in care, are recommended. Further in-depth qualitative research is recommended to explore adherence history and general health-seeking behaviours of people living with HIV, to provide explanations for failure on second-line ART regimens.

## Figures and Tables

**Table 1:** Socio-demographic and clinical characteristics by viral re-suppression of PLHIV on second-line antiretroviral therapy in Bulawayo, January 2015–December 2020 (N = 315).

Categories		Viral Load (in Copies RNA/mL)				*p*-Value
		<1000		≥1000		
	N	n	%	n	%	
**Total**	315	216	68.6	99	31.4	
**Gender**						0.687
Female	158	110	69.6	48	30.4	
Male	157	106	67.5	51	32.5	
**Age (in years)**						0.841
Child (<15)	22	17	77.3	5	22.7	
Adolescent (15–19)	41	28	68.3	13	31.7	
Young Adult (20–24)	56	38	67.9	18	32.1	
Adult (>24)	196	133	67.9	63	32.1	
**Marital status (>18 years)**						0.110
Married	79	57	72.2	22	27.8	
Single	173	114	65.9	59	34.1	
Separated	10	4	40.0	6	60.0	
Divorced	10	9	90.0	1	10.0	
**Educational status**						0.637
No education	96	69	71.9	27	28.1	
Primary	63	45	71.4	18	28.6	
Secondary	139	90	64.7	49	35.3	
Tertiary	17	12	70.6	5	29.4	
**Employment status (>18 years)**						0.512
Employed	47	34	72.3	13	27.7	
Unemployed	225	150	66.7	75	33.3	
**WHO clinical stage**						0.091
I & II	143	105	73.4	38	26.6	
III & IV	172	111	64.5	61	35.5	
**EAC sessions**						0.010 *
3–6	204	150	73.5	54	26.5	
>6	111	66	59.5	45	40.5	
**Baseline CD4 count (cells/µL)**						0.039 *
≤200	102	62	60.8	40	39.2	
>200	213	154	72.3	59	27.7	
**Viral load at switch (copies/mL)**						0.046 *
≤5000	87	67	77.0	20	23.0	
>5000	228	149	65.4	79	34.6	
**First-line drug substitution history**						0.143
Yes	140	90	64.3	50	35.7	
No	175	126	72.0	49	28.0	
**First-line drugs**						0.527
ABC/3TC/EFV	7	4	57.1	3	42.9	
ABC/3TC/NVP	9	4	44.4	5	55.6	
AZT/3TC/EFV	9	7	77.8	2	22.2	
AZT/3TC/NVP	62	48	77.4	14	22.6	
D4T/3TC/EFV	5	3	60.0	2	40.0	
D4T/3TC/NVP	152	104	68.4	48	31.6	
TDF/3TC/EFV	60	39	65.0	21	35.0	
TDF/3TC/NVP	11	7	63.6	4	36.4	
**Second-line drugs**						0.093
ABC/3TC/ATZ/rtv	81	52	64.2	29	35.8	
ABC/3TC/DTG	4	2	50.0	2	50.0	
ABC/3TC/LOP/rtv	40	30	75.0	10	25.0	
AZT/3TC/ATZ/rtv	64	42	65.6	22	34.4	
AZT/3TC/DTG	3	0	0.0	3	100.0	
AZT/3TC/LOP/rtv	11	7	63.6	4	36.4	
AZT/3TC/NVP	3	2	66.7	1	33.3	
D4T/3TC/LOP/rtv	1	1	100.0	0	0.0	
TDF/3TC/ATZ/rtv	67	52	77.6	15	22.4	
TDF/3TC/DTG	22	12	54.5	10	45.5	
TDF/3TC/LOP/rtv	19	16	84.2	3	15.8	
**Duration on first-line ART (in years)**						0.005 *
≤5	145	111	76.6	34	23.4	
>5	170	105	61.8	65	38.2	
**History of non-adherence**						<0.001 *
Yes	180	100	55.6	80	44.4	
No	135	116	85.9	19	14.1	
**Body Mass Index (kg/m^2^)**						0.085
≥18.5	233	166	71.2	67	28.8	
<18.5	82	50	61.0	32	39.0	
**Time between failure and drug switch (months)**						<0.001 *
3–12	125	109	87.2	16	12.8	
>12	190	107	56.3	83	43.7	
**Retention in care**						<0.001 *
Yes	263	195	74.1	68	25.9	
No	52	21	40.4	31	59.6	

N = 315; Categories > 18 years N = 272; *, denotes statistical significance at *p* < 0.05; d4T, Stavudine; TDF, Tenofovir; ABC, Abacavir; AZT, Zidovudine; 3TC, Lamuvidine; EFV, Efavirenz; NVP, Nevirapine; ATZ/rtv, Atazanavir/Ritonavir; DTG, Dolutergravir; LOP/rtv, Lopinavir/Ritonavir; EAC, Enhanced Adherence Counselling; ART, antiretroviral therapy.

**Table 2:** Determinants of viral re-suppression among PLHIV on second-line therapy in Bulawayo Zimbabwe, January 2015–December 2020 (n = 315).

Categories	Crude OR	95% CI	Adjusted OR	95% CI
**EAC sessions**				
>6	1	-	1	-
3–6	1.89	1.16–3.09	1.63	0.89–2.97
**Baseline CD4 count (cells/µL)**				
≤200	1	-	1	-
>200	1.68	1.02–2.77	**1.94**	**1.04–3.60**
**Viral load at switch (copies/mL)**				
>5000	1	-	1	-
≤5000	1.77	1.00–3.13	1.13	0.56–2.29
**Duration on first-line ART (in years)**				
≥5	1	-	1	-
<5	2.02	1.23–3.31	1.04	0.56–1.93
**History of non-adherence**				
Yes	1	-	1	-
No	4.88	2.77–8.61	**3.88**	**1.91–7.85**
**Time between failure and drug switch (in months)**				
>12	1	-	1	-
3–12	5.28	2.90–9.60	**4.13**	**1.98–8.60**
**Retention in care**				
No	1	-	1	-
Yes	6.30	2.84–13.99	**6.35**	**2.56–15.76**

Note: Bold data shows significance at 5% significance level. OR, odds ratio; 95% CI, 95% confidence interval; EAC, Enhanced Adherence Counselling; ART, antiretroviral therapy.

## Data Availability

The dataset extracted and analysed in this study are not publicly available as the Zimbabwe Ministry of Health and Child Care is the custodian of all routine patient-level data. Data are available from the corresponding author on reasonable request.
